# Rare DDX3X Gene Mutation in a Male Newborn With Super-refractory Status Epilepticus Responding to Lacosamide Drug Therapy

**DOI:** 10.7759/cureus.75572

**Published:** 2024-12-11

**Authors:** Noora BaniHammad, Raafat Hamad Seroor H Jadah

**Affiliations:** 1 Medicine and Surgery, Bahrain Defence Force Hospital, Riffa, BHR; 2 Pediatric Neurology, Bahrain Defence Force Hospital, Riffa, BHR

**Keywords:** epileptic seizures, genetic syndromes, lacosamide, neonatal neurology, seizure medications

## Abstract

Super-refractory status epilepticus (SRSE) is defined as status epilepticus that persists or recurs after treatment with anesthetic agents for more than 24 hours, including cases with recurrent seizures on reduction or withdrawal of anesthetic drugs. Super-refractory status epilepticus presents a significant challenge for neurologists, particularly when standard treatments fail to achieve seizure control. Lacosamide, which has a unique mechanism involving modulating voltage-gated sodium channels by enhancing their slow inactivation, has emerged as a potential option for managing SRSE.

We report a case of a male neonate with SRSE who failed to respond to first-line and second-line antiepileptic drugs (AEDs), including benzodiazepines, phenobarbital, levetiracetam, and midazolam infusion. Following an extensive review of the available treatment options, lacosamide was introduced as a third-line agent. When lacosamide was introduced to the treatment regimen, it led to a significant reduction in seizure frequency and ultimately achieved seizure control. Lacosamide was well tolerated by the patient with no significant side effects.

Upon further assessment of the patient using whole exome sequencing (WES), it was elucidated that he is a carrier of the *DDX3X* gene mutation, which is known as syndromic X-linked intellectual developmental disorder of the Snijders Blok type (MRXSSB). This syndrome is characterized by global developmental delay, intellectual disability, behavioral disorders, and seizures. However, the neonate described in our case report presents in an atypical manner in comparison to those with a *DDX3X* gene mutation.

This case highlights lacosamide's potential role in the management protocol of SRSE, particularly in neonatal patients with MRXSBB who are unresponsive to standard therapies. It is important to shed light on the possibility of using lacosamide in SRSE in neonates. However, further studies are needed to establish lacosamide's efficacy and safety profile more comprehensively. This case contributes to the growing body of evidence supporting lacosamide's use in difficult-to-treat seizure disorders.

## Introduction

Super-refractory status epilepticus (SRSE) is a severe neurological condition, defined as status epilepticus that continues for 24 hours or more despite treatment with anesthetic agents [1]. SRSE poses significant difficulties, especially in neonates, where there are fewer therapies and guidelines are lacking. Effective management of SRSE is crucial since prolonged seizure activity can lead to permanent neurological disability.

The incidence of neonatal super-refractory status epilepticus is not well established, with limited data in the neonatal population. Status epilepticus occurs in 16%-25% of neonates with seizures [2,3]. Estimates suggest that up to 10%-40% of neonatal status epilepticus cases may become refractory. As for super-refractory status epilepticus, there are no precise statistics available for its incidence in neonates due to the limited number of cases reported and the variability in defining SRSE across studies.

Lacosamide is a promising antiepileptic drug (AED) that can be implemented in the management of super-refractory status epilepticus in children and neonates. It functions by focusing on the voltage-gated sodium channel's slow activation mechanism, maintaining the integrity of hyperexcitable neuronal membranes, and reducing the sustained repetitive firing of neurons that precedes seizures [4,5]. A rising number of research and case reports point to lacosamide's potential use as an adjunct therapy in SRSE situations where conventional AEDs are ineffective [6-9].

Recent case studies have demonstrated lacosamide's effectiveness in SRSE, showing encouraging results in the treatment of children and neonates who did not respond to the traditional first- and second-line treatments. However, studies about the use of lacosamide in SRSE are rare, and its use in neonatal patients with SRSE is even rarer [7,10].

Furthermore, there are no documented case reports regarding the use of lacosamide in the context of syndromic X-linked intellectual developmental disorder of the Snijders Blok type (MRXSSB). This condition is characterized by intellectual disability, delayed language development, and distinctive facial features, including a prominent forehead, hypertelorism, and a wide nasal bridge. In neonates, MRXSSB may present with early developmental delays, but clear syndromic features might not be immediately evident, often making it challenging to diagnose at birth. Refractory seizures are also associated with this condition, although they may not appear until later in infancy or childhood. These seizures can be difficult to control and may contribute to the complexity of the clinical management, potentially worsening developmental outcomes and quality of life for affected children [11,12].

The neonate with MRXSSB and SRSE described in this case report did not respond to traditional first- and second-line anticonvulsants, including benzodiazepines, phenobarbital, levetiracetam, and midazolam. Remarkably, the use and administration of lacosamide resulted in a sizable reduction in seizure activity and ultimately achieving seizure control. This report provides a vulnerable population with better therapy options and adds to the mounting body of evidence supporting the off-label use of lacosamide in neonates with MRXSSB and SRSE.

## Case presentation

We describe a case of a newborn male neonate who presented to the emergency department on the second day of life with complaints of periodic abnormal repeated myoclonic jerks involving the head and limbs, lasting for a few seconds, and increasing in duration since his delivery. There was no history of fever, vomiting, cyanosis, poor feeding, or reduced activity.

The neonate was born at full term through a normal delivery without any complications. His physical examination showed no dysmorphic features, apart from polydactyl of a single right digit. There were no neurocutaneous stigmata, he had positive primitive reflexes, and he had normal tone, power, and deep tendon reflexes. Additionally, he had a normal cranial nerve examination, other systemic examinations were unremarkable, and there was no family history of seizures or epilepsy.

The patient was admitted to the hospital and started on the status epilepticus protocol with initial short-acting benzodiazepine (IV diazepam 0.2 mg/kg) twice followed by a loading dose of phenytoin 20 mg/kg. However, he continued to seize, so he was started on IV phenobarbital with a total dose of 40 mg/kg, followed by IV levetiracetam with a total dose of 40 mg/kg. Despite this aggressive management, we were unsuccessful in achieving control of these myoclonic seizures.

Correspondingly, the patient was then shifted to the intensive care unit (ICU) where he was intubated and ventilated, and a midazolam infusion was started with an initial dose of 1 mcg/kg/minute, which was increased gradually up to 12 mcg/kg/minute during his hospital course. Despite this, the patient continued to seize, so a decision was made to start IV lacosamide at a loading dose of 10 mg/kg, followed by a maintenance dose that was started initially at 1 mg/kg/day, which was gradually increased to 12 mg/kg/day. The patient showed a good response after 48 hours of starting lacosamide, and there was no further seizure activity.

Moreover, following a period of observation, the midazolam infusion was gradually titrated and ultimately stopped, and the patient was then discharged on lacosamide 12 mg/kg/day and levetiracetam 60 mg/kg/day. He was given a follow-up appointment with the pediatric neurologist to monitor his development, intellect, behavior, and further seizure activity.

**Figure 1:**
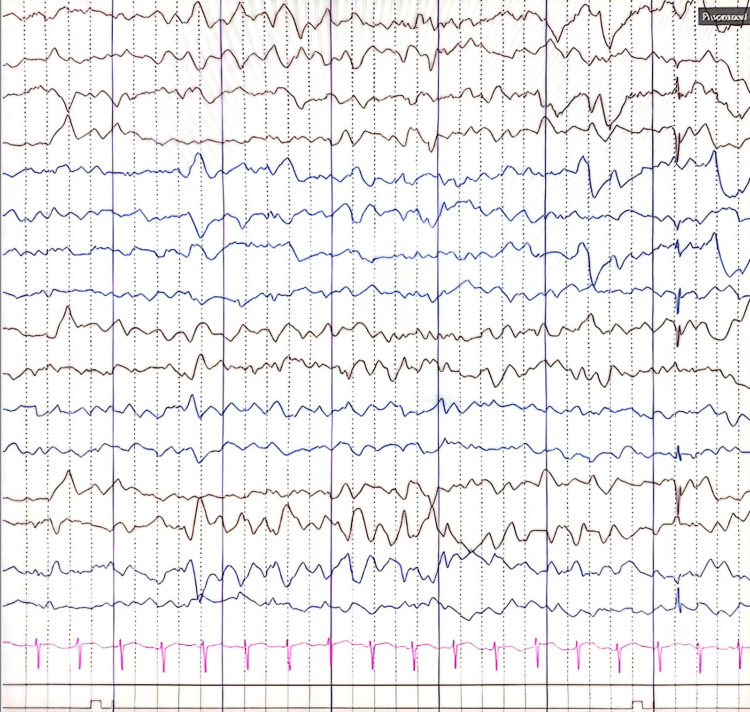
Multifocal epileptiform spikes and polyspikes with discharges EEG settings: baby montage, sensitivity 7 microvolt, high filter settings (70 Hz) EEG: electroencephalogram

His initial laboratory tests, including a complete blood count and biochemistry, were unremarkable. An electroencephalogram (EEG) was done when the patient initially presented to the hospital on day 2 of life, and it was abnormal, showing bi-centro-temporal epileptiform spike and polyspike wave discharges along with central regions seizure activity (Figure 1). A brain magnetic resonance imaging (MRI) was also done, and it was unremarkable. Our patient also underwent a lumbar puncture, and the cerebrospinal fluid (CSF) study for neurotransmitters was negative.

Whole exome sequencing (WES) was done, and the result was positive for a *DDX3X* gene mutation, specifically, a hemizygous pathogenic variant chrX_41342643_C/Tc.433C>T, P.Arg145Cys NM_001356.5. This is correlated with syndromic X-linked intellectual developmental disorder of the Snijders Blok type (MRXSSB).

## Discussion

Status epilepticus is a neurological emergency that requires urgent recognition and treatment as it is associated with significant morbidity and mortality. Super-refractory status epilepticus (SRSE) is a severe and prolonged form of status epilepticus, characterized by seizures that continue or recur despite treatment with anesthetic agents for 24 hours or more [1,9]. This definition also encompasses cases where seizures persist even after the reduction or withdrawal of anesthesia, making it a life-threatening condition that necessitates prolonged and intensive care interventions. SRSE is particularly difficult to manage, requiring the use of advanced and, in some cases, experimental therapies beyond the usual antiepileptic drugs (AEDs) used in the initial stages of treatment.

The exact incidence of SRSE in neonates remains poorly defined due to the rarity of the condition and limited studies specific to this age group. Neonatal seizures are relatively common, occurring in approximately 1-5 per 1,000 live births, with around 16%-25% of these cases progressing to status epilepticus [2]. Of these, a subset may develop refractory status epilepticus (RSE), with an estimated 7.14% failing to respond to first-line and second-line treatments [2,5].

In neonates, the etiology of SRSE is often linked to severe underlying brain insults or genetic predispositions. Some of the common causes include hypoxic-ischemic encephalopathy (HIE), which is the most common cause of neonatal seizures, where perinatal asphyxia leads to brain injury. Other causes include intracranial hemorrhage and genetic or metabolic disorders such as *SCN1A* mutations. Cerebral malformations and infections are also common causes of SRSE. Given the heterogeneity of these causes, management strategies are highly individualized, focusing both on seizure control and addressing the underlying condition [3,6].

In this case, the underlying cause of the SRSE could be related to the *DDX3X* gene mutation that was identified in the patient. Although the neonate did not present with the typical features of MRXSSB such as macrocephaly, brain abnormalities, and global developmental delay, he presented with SRSE. Currently, there are 63 reported cases in the literature of syndromic X-linked intellectual developmental disorder of the Snijders Blok type, and of those cases, 13% had seizures, but none of the cases reported super-refractory status epilepticus [11]. Additionally, individuals with Snijders Blok-Campeau syndrome (SNIBCPS) commonly have brain abnormalities, which predispose them to seizures. However, in this case, the neonate had no dysmorphic features and no brain abnormalities that were evident on imaging.

The *DDX3X* gene mutation can result in seizure activity due to its underlying role in neuronal function. This is because the *DDX3X* gene encodes for a DEAD-box RNA helices, which is involved in regulating RNA processing, translation, and stress response. Specifically, mutations in this gene can disrupt neuronal development and connectivity, leading to conditions for hyperexcitable neural networks. Additionally, impairments in mRNA translation regulation at synapses can alter neurotransmitter balance, which would trigger seizure activity. Thus, these changes highlight the gene's critical role in maintaining neuronal stability and its potential to cause seizures when disturbed [11].

The management of neonatal SRSE commonly involves a combination of antiepileptic drugs and anesthetics. First-line antiseizure medications include benzodiazepines such as midazolam, lorazepam, and diazepam. These are often followed by second-line agents such as phenytoin, fosphenytoin, phenobarbitone, levetiracetam, propofol, and sodium thiopental. Midazolam is commonly used for refractory seizures, typically delivered as a continuous infusion in SRSE. In cases where seizures persist, anesthetic therapies such as ketamine may be considered. Despite these therapies, SRSE remains challenging to control in neonates, necessitating individualized, aggressive treatment plans [8-10].

Lacosamide is an antiepileptic drug that acts by enhancing the slow inactivation of voltage-gated sodium channels, stabilizing hyper-excitable neuronal membranes. Although its use in neonates remains off-label, there have been emerging case reports that suggest lacosamide may be a useful adjunctive treatment in SRSE when conventional therapies fail. Its unique mechanism of action differs from other sodium channel blockers, which act on fast inactivation, potentially offering a new therapeutic avenue for managing drug-resistant seizures [4].

This case demonstrates the efficacy of lacosamide as an add-on therapy for the management of SRSE. This is evident as the patient was trailed on multiple different antiepileptic regimens; however, the seizures only showed improvement after the use of lacosamide. Similarly, a case report by Bertozzi et al. highlighted the efficacy of lacosamide in a neonate who developed SRSE following GBS meningoencephalitis [7], whereby lacosamide was started as an adjunctive therapy to phenobarbital, phenytoin, and continuous infusion of ketamine and midazolam. Following this, there was an improvement in the clinical condition of the neonate, and this persisted even after the suspension of all medications, except for phenobarbital. The patient remained seizure-free after the introduction of lacosamide and is being followed up regularly by the pediatric neurologist to monitor for further seizure activity.

Similarly, a study by Arkilo et al. supported the findings that were seen in our case study [8]. It was the first retrospective study that looked at children under 12 years of age receiving IV lacosamide for refractory status epilepticus. The patients in their study showed better seizure control when concurrently using lacosamide and phenytoin. This suggests that when used with other drugs, lacosamide might be more effective due to its synergistic effect. In our case, the patient was receiving midazolam infusions when we administered lacosamide intravenously, which resulted in decreasing seizure activity, potentially due to its synergistic effects as in the study by Arkilo et al. [8].

Furthermore, the prognosis of super-refractory status epilepticus in neonates is generally poor and depends largely on the underlying etiology. Neonates with SRSE often face significant risks of long-term neurological sequelae, including cognitive impairment, motor deficits, and epilepsy, particularly in cases where the underlying cause is structural brain damage or a severe genetic disorder. Mortality rates are high in neonatal SRSE, ranging from 30% to 50%, depending on the cause and the timeliness of intervention. Survivors frequently experience neurodevelopmental delays and may have persistent epilepsy that requires long-term management. Early recognition, aggressive treatment, and individualized care strategies are crucial for improving outcomes [9].

## Conclusions

The successful use of lacosamide in our case report not only highlights its potential as an effective treatment option for SRSE in neonates. It also underscores the need for further research, especially regarding the *DDX3X* genetic abnormality and its relation to SRSE. Disruptions in the *DDX3X* gene can alter neural differentiation, synaptic activity, and overall brain homeostasis, predisposing individuals to seizure activity. Larger studies and clinical trials are necessary to establish both the role of lacosamide in SRSE management definitively and the role of lacosamide in the management of SRSE related to MRXSSB. The case presented herein demonstrates the potential efficacy of lacosamide in achieving seizure control in a neonate with MRXSSB SRSE who did not respond to conventional treatments. Notably, the patient experienced a significant reduction in seizure frequency and improvement in neurological status following the introduction of lacosamide.
